# Supplementary figures and images for: Phylogenetic profiling in eukaryotes: The effect of species, orthologous group, and interactome selection on protein interaction prediction
Source: PLoS One. 2022 Apr 14;17(4):e0251833. doi: 10.1371/journal.pone.0251833 (PMC9009711; doi:10.1371/journal.pone.0251833)

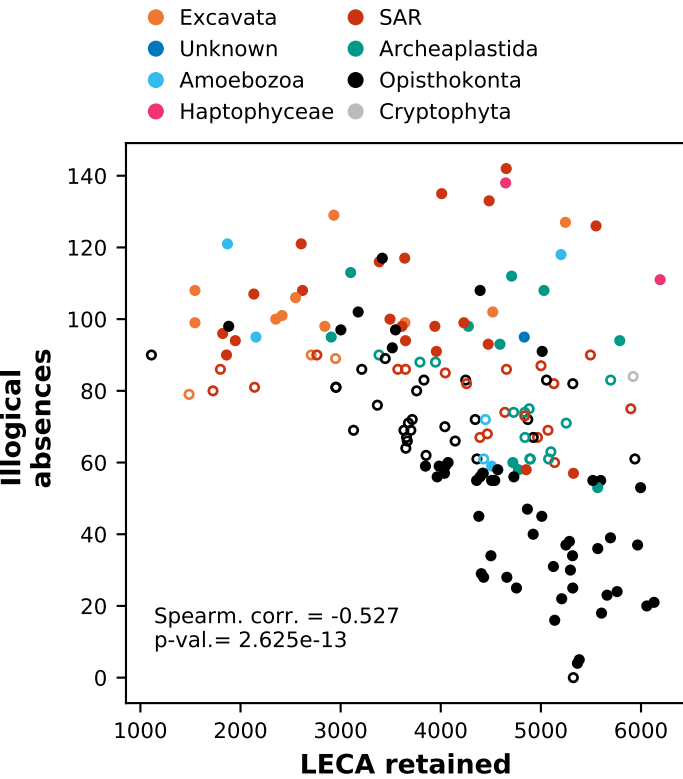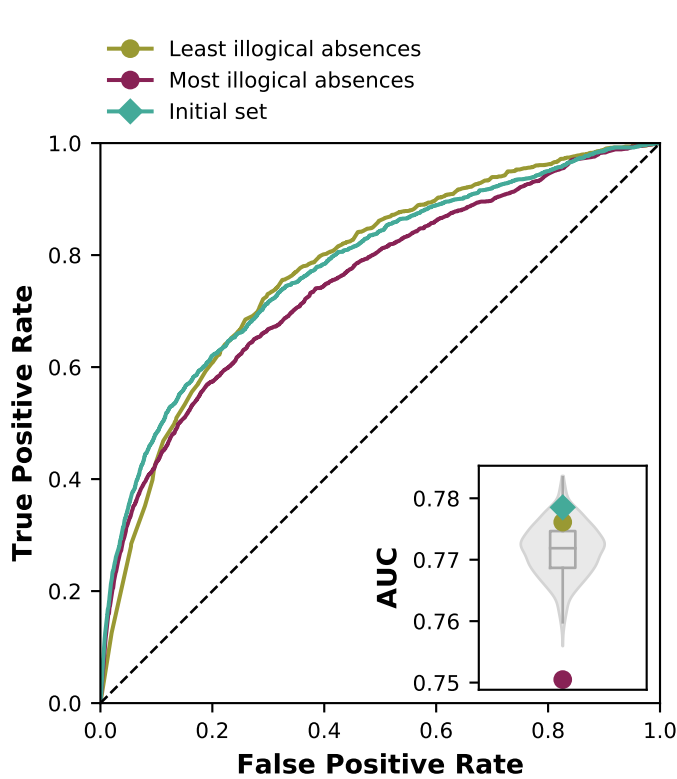

Supplement: S1 Fig — A. Illogical absences as a function of retained LECA OGs in different species. We see that where most Opisthokonta scores similarly low with the BUSCO metric, they score lower with the IA metric indicating a difference between the two metrics. However, the performance of genomes selected with both metrics are similar to each other. Filled data points are the selected genomes for the prediction accuracy calculations. B. Receiver-operator Curve of two species sets (n = 50) with the most and least illogical absences. The inset gives the Area Under the Curve (AUC) values compared with the random backdrop of 1000 random species sets (violin plot) and the initial species set (teal diamond). Human has a perfect score of 0 illogical absences since the interactions are from the human reference interactome. Therefore, we did not select human for the genome set. (PDF) [file pone.0251833.s001.pdf]

**A.**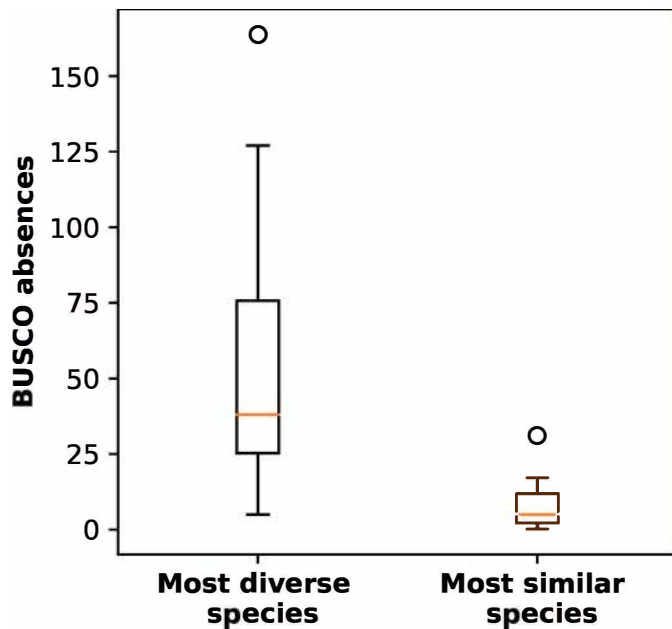**B.**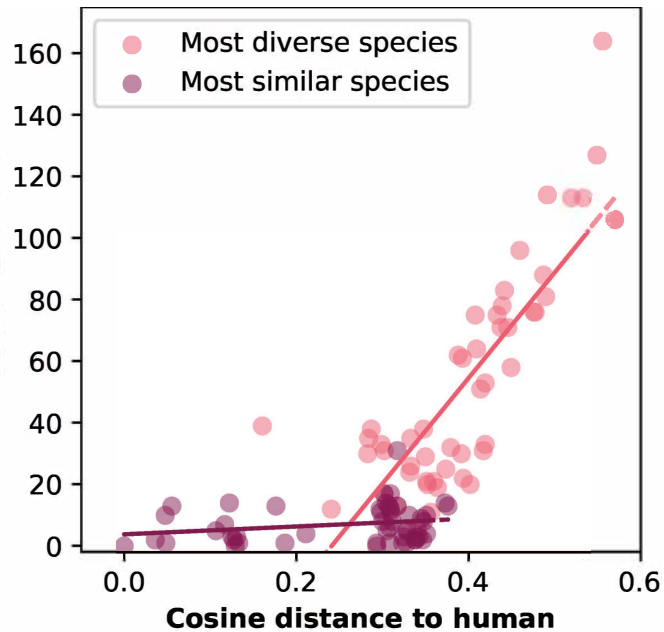

Supplement: S4 Fig — A. BUSCO absences in the most diverse species set are higher than in the most similar species set. B. The BUSCO absences vs. the cosine distance to human shows that most similar species set is more similar to the human genome, and likely enriched with high quality Opisthokonta genomes (as can also be seen in Fig 3A). (PDF) [file pone.0251833.s004.pdf]

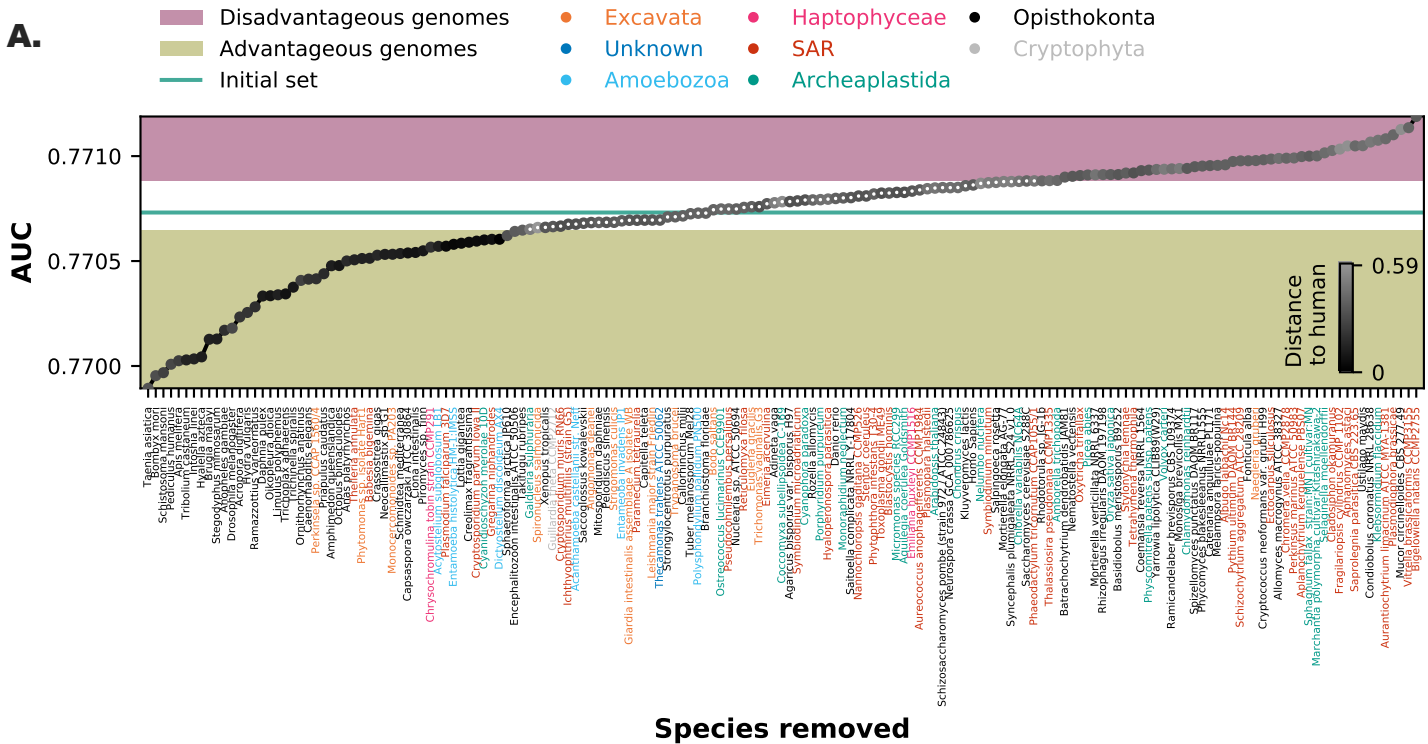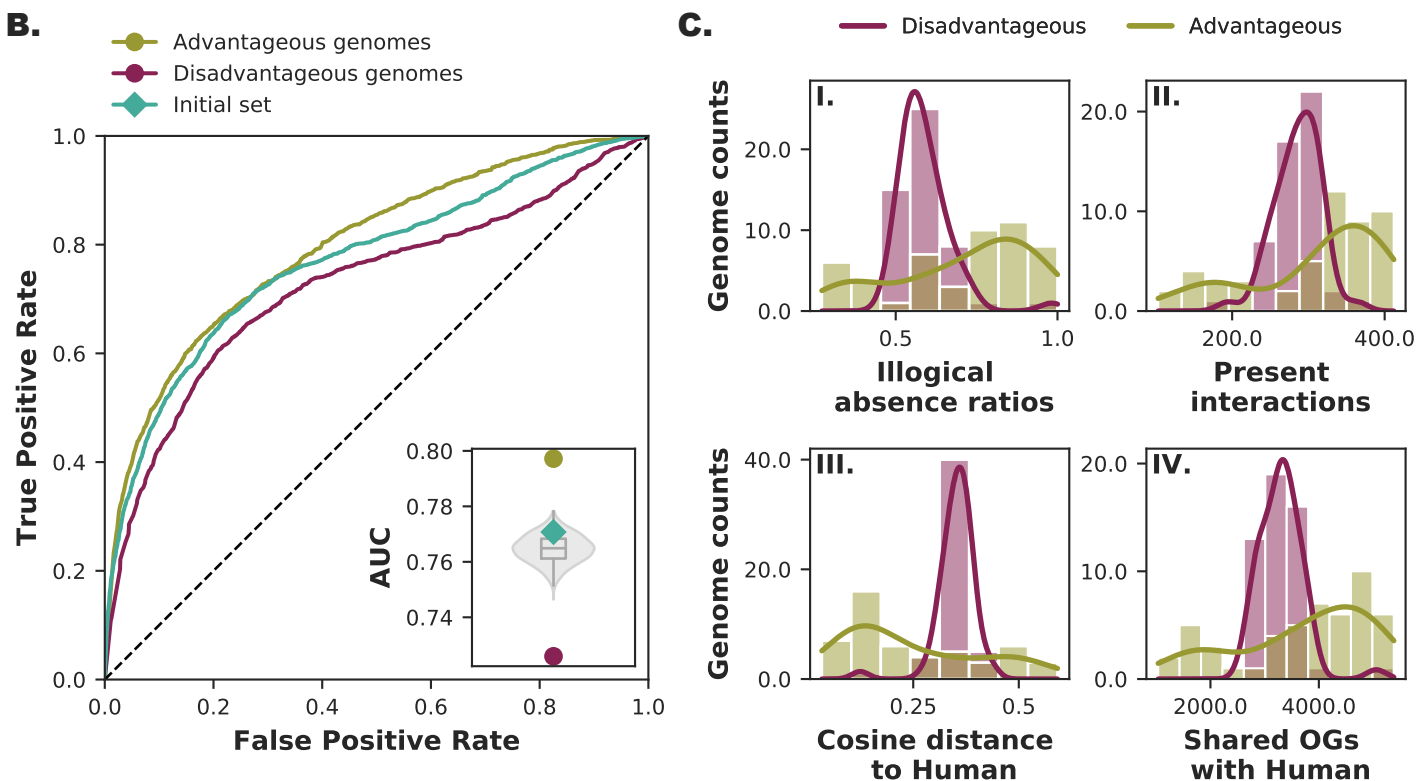

Supplement: S5 Fig — A. Recalculated Area Under the Curve (AUC) values when a single species is removed from the initial species set. Genomes that increase the AUC value when removed can be considered disadvantages compared to the initial set when predicting protein interactions with phylogenetic profiles. Genomes that decrease the AUC value when removed can be considered advantageous for predicting protein interactions. Top 50 advantageous and top 50 disadvantageous genomes shown with the black fill in the scatter plot. B. Receiver-operator Curve of two species sets (n = 50) with the most advantageous and disadvantageous genomes. The inset gives the Area Under the Curve (AUC) values compared with the random backdrop of 1000 random species sets (violin plot) and the initial species set (green diamond). C. Comparison of the counts (histogram) and kernel density estimates (line plot) of (I) illogical absence ratios (illogical absences divided by total interaction absences (co-absences + illogical absences)), (II) present interactions, (III) the cosine distance to human, and (IV) total shared OGs with human. (PDF) [file pone.0251833.s005.pdf]

**A.**

● Advantageous    ● Disadvantageous

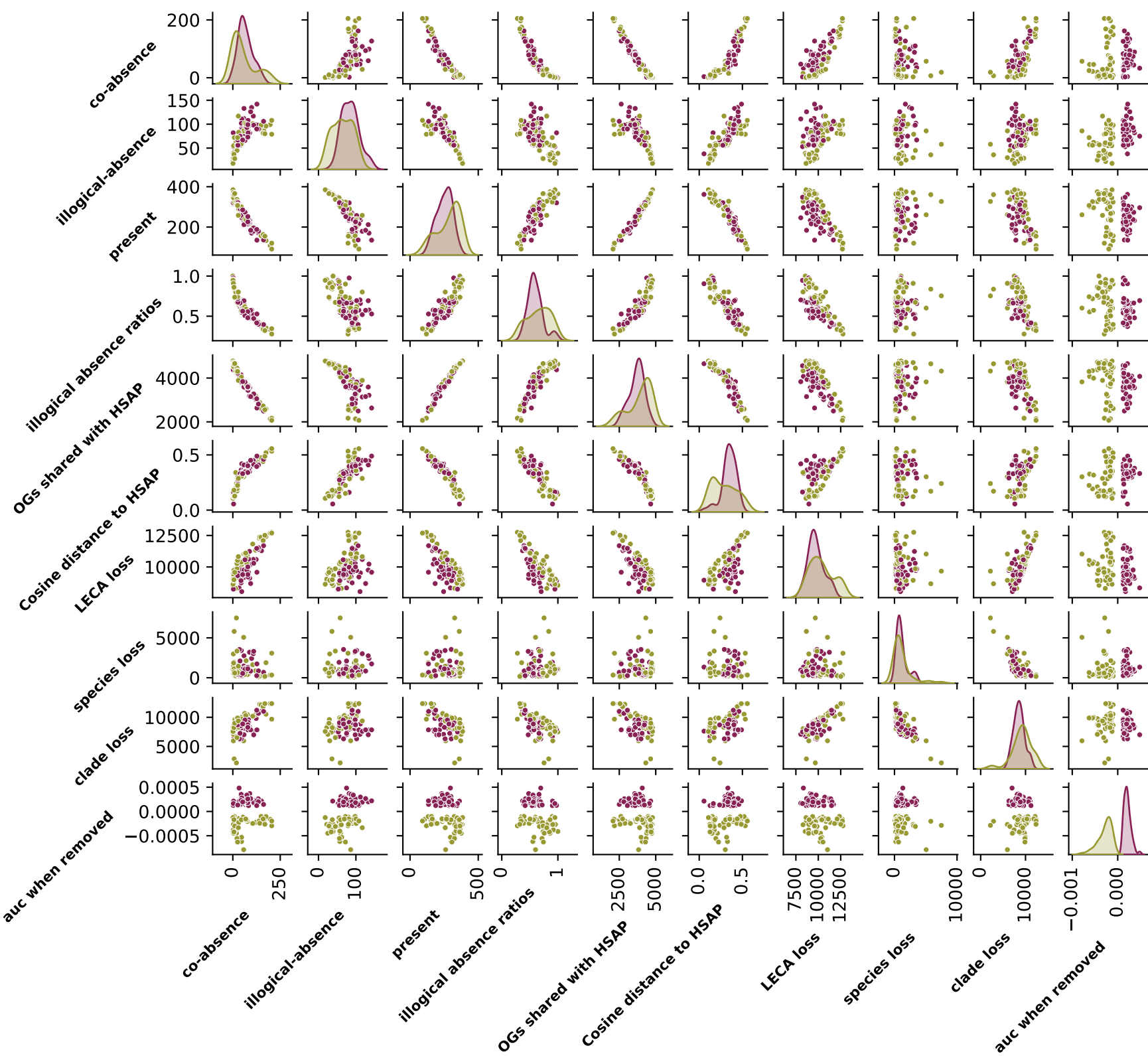

**B.**

● Advantageous ● Disadvantageous

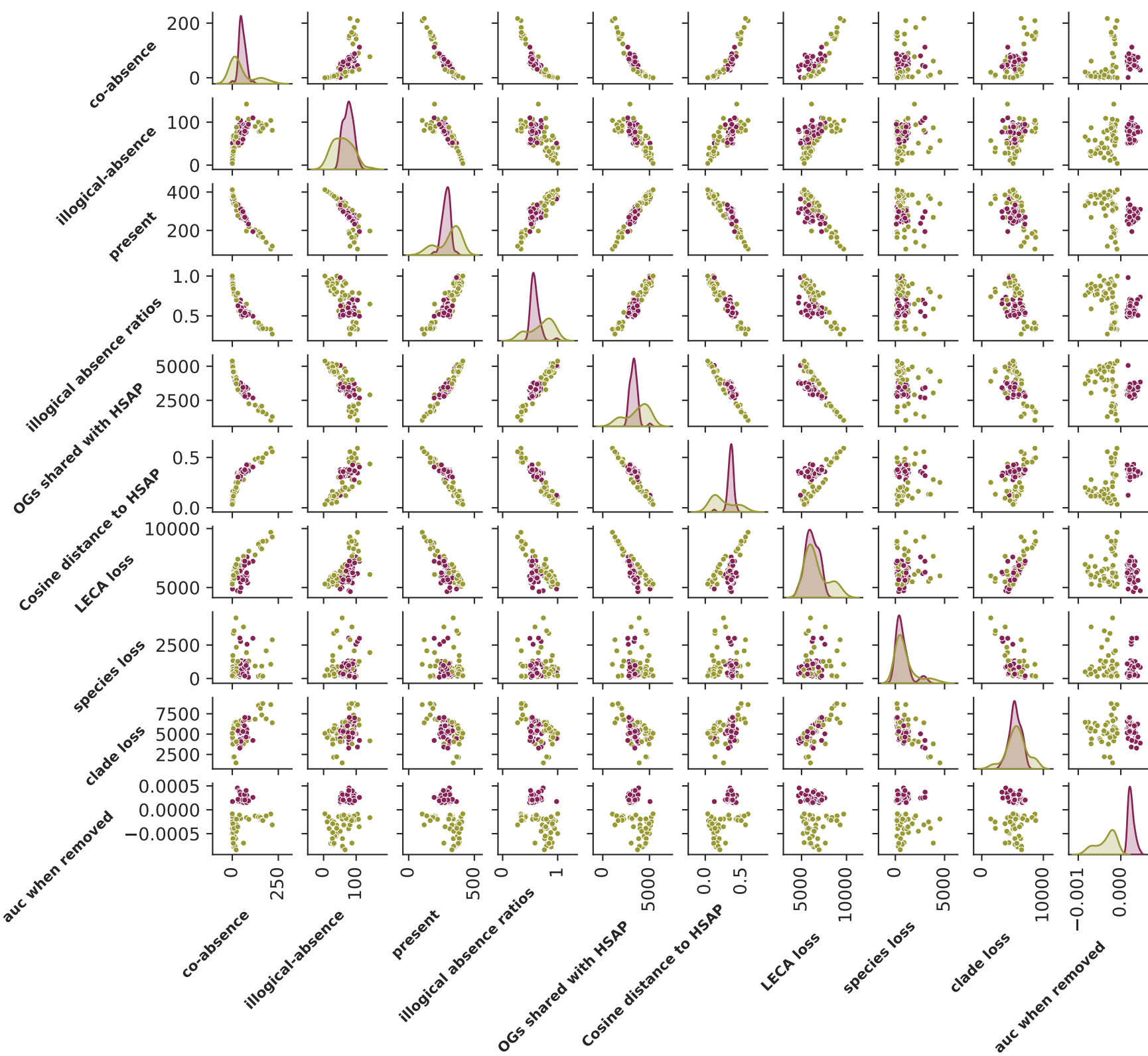

Supplement: S6 Fig — Given for A. Sonicparanoid and B. Broccoli inferred OGs. From top to bottom (or left to right) the interactions that are co-absent; illogically absent; and present; the ratio of illogical absences to total absences; number of OGs shared with the human genome; the cosine distance to the human genome; LECA OGs loss (Dollo parsimony inferred); species (lineage) specific loss; (clade) ancestral loss; and the difference in AUC from the initial set AUC when a genome is removed. (PDF) [file pone.0251833.s006.pdf]

**A.**

LECA OGs

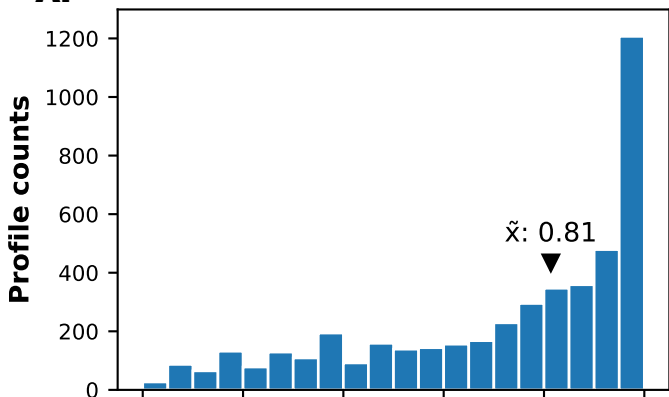**B.**

LECA OGs

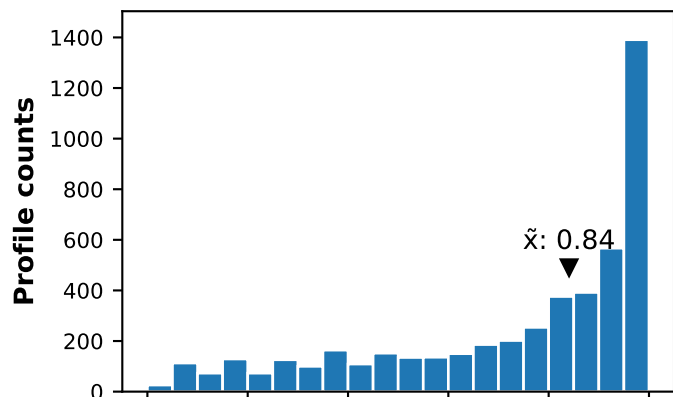

post-LECA OGs

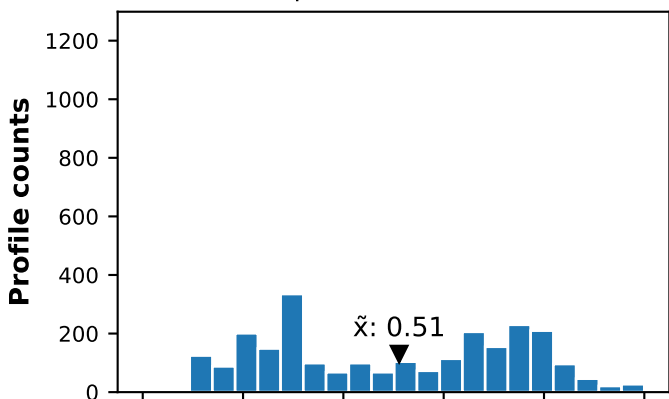

post-LECA OGs

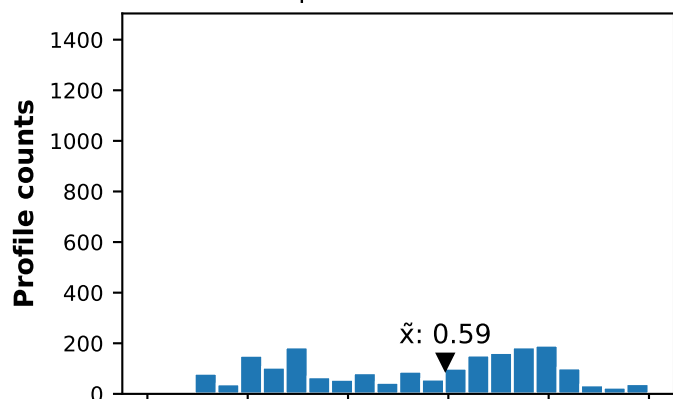

All OGs

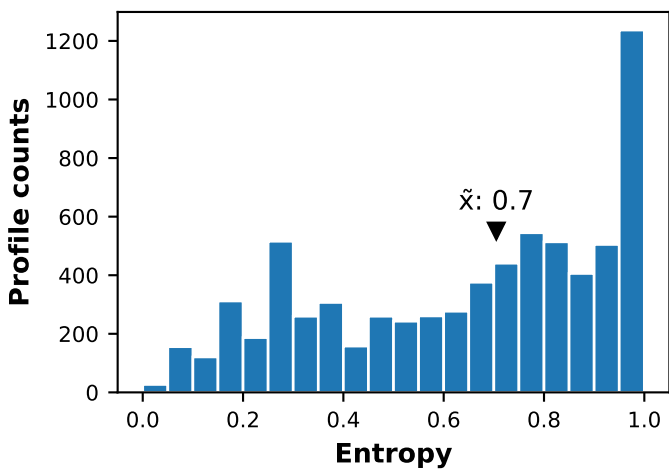

All OGs

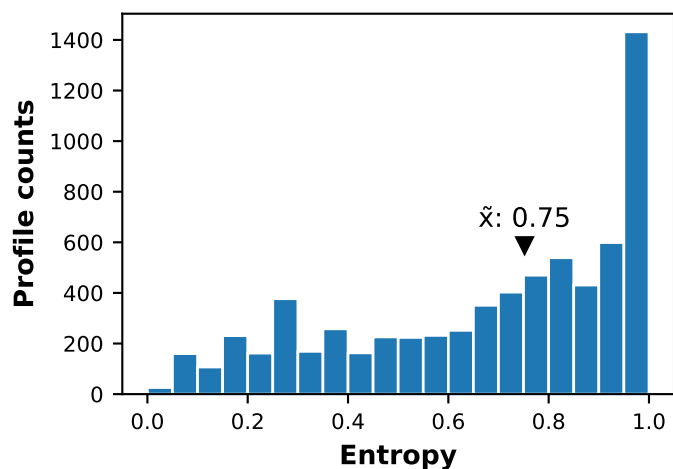

Supplement: S7 Fig — Given for A. Sonicparanoid and B. Broccoli inferred OGs. From top to bottom, the entropy is shown in profiles for LECA, post-LECA and all OGs. Median entropy is presented with a black arrow. Mann-Whitney U test shows significant difference between distributions of LECA, post-LECA and all OGs, p-value < 0.001. (PDF) [file pone.0251833.s007.pdf]

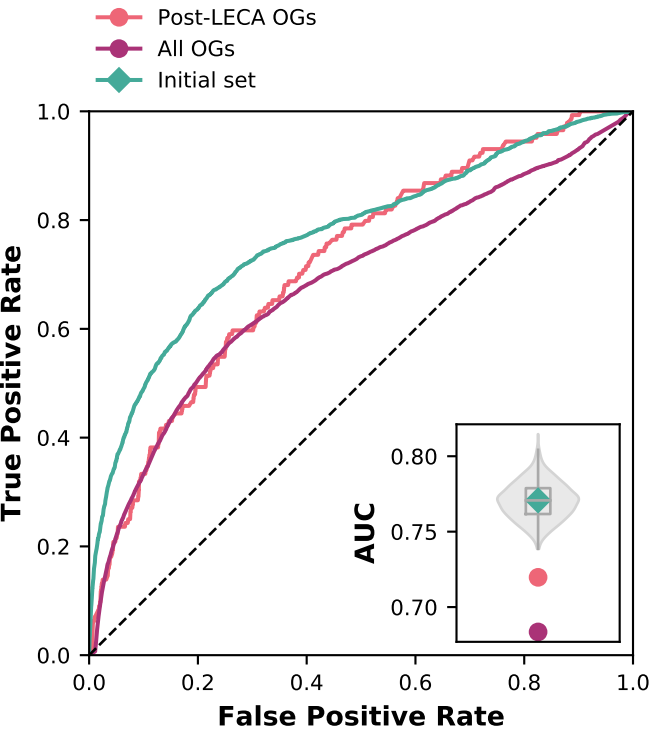

Supplement: S8 Fig — Receiver-operator Curve of post-LECA OGs and unfiltered OGs. The inset gives the Area Under the Curve (AUC) values compared with the random backdrop of randomly selected LECA OGs (violin plot) and the initial species set (green diamond). (PDF) [file pone.0251833.s008.pdf]

**A.**

LECA (initial) set    Post-LECA OGs

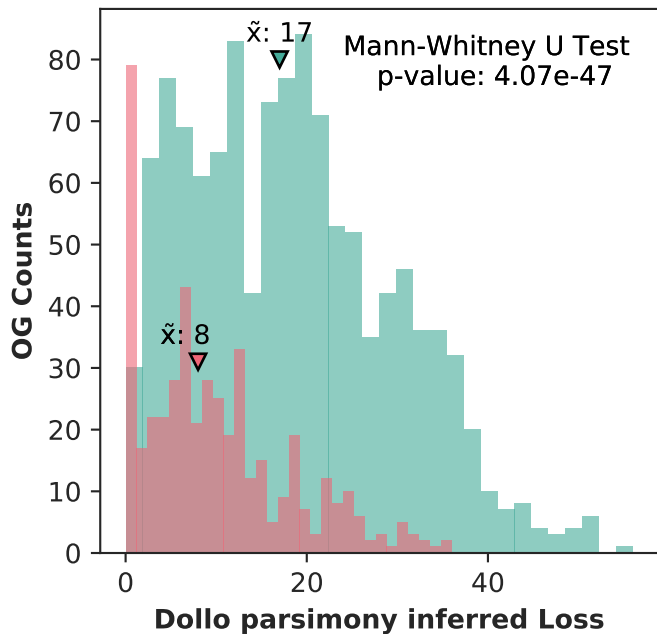**B.**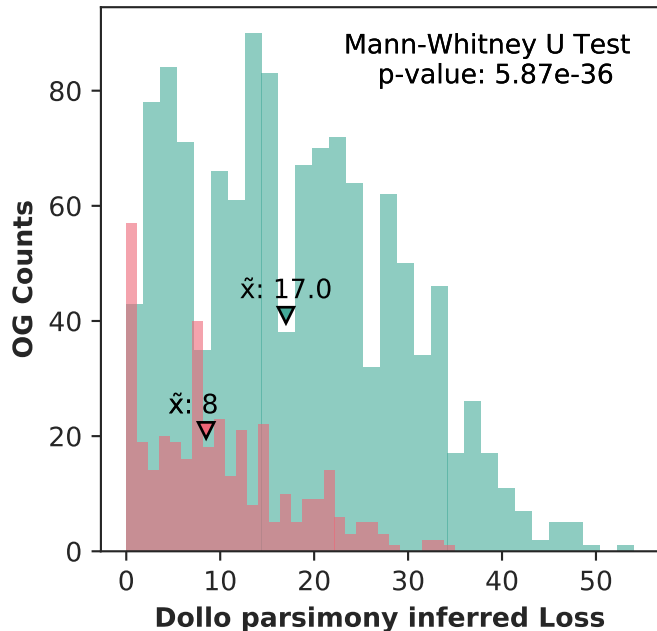

Supplement: S9 Fig — Given for A. Sonicparanoid and B. Broccoli inferred OGs. Mann-Whitney U test shows significant difference between distributions. (PDF) [file pone.0251833.s009.pdf]

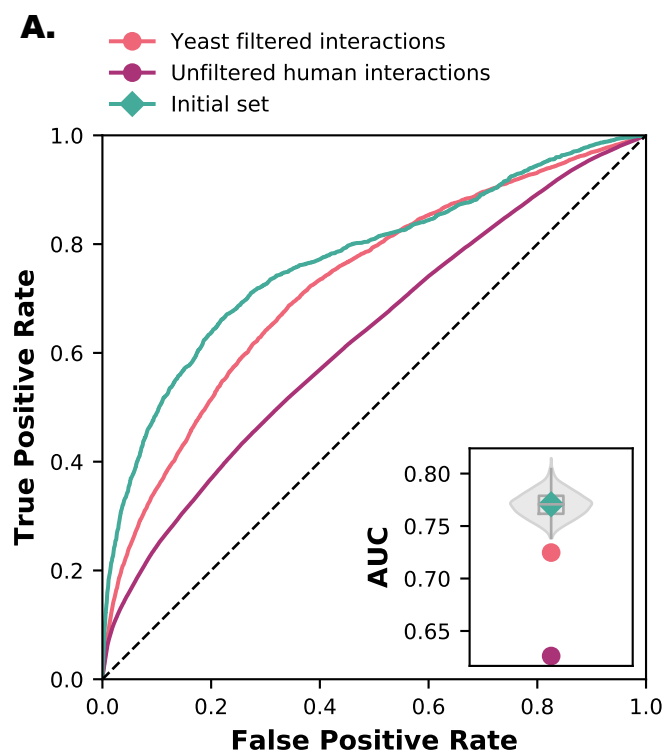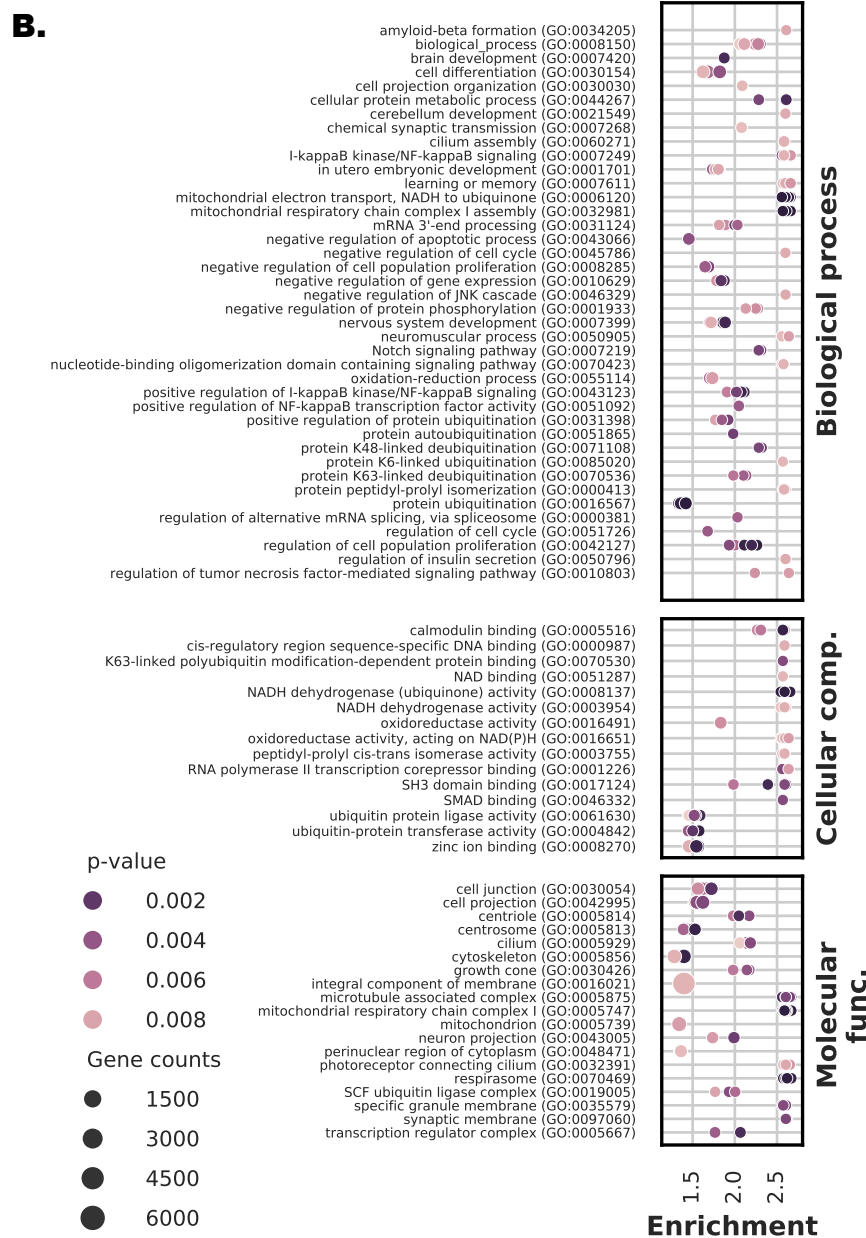

Supplement: S11 Fig — A. Receiver-operator Curve of post-LECA and unfiltered OGs of Broccoli. The inset gives the Area Under the Curve (AUC) values compared with the random backdrop of randomly selected LECA OGs (violin plot) and the initial species set (green diamond). B. GO-enrichment analysis for genes enriched in interactions present in only human vs. interactions present in human and yeast. OGs can contain multiple genes. We randomly selected genes from an OG to generate new sample and population sets 10 times and recalculated the enrichment (shown by multiple points in the figure rows). (PDF) [file pone.0251833.s011.pdf]

**A.**

Human Yeast

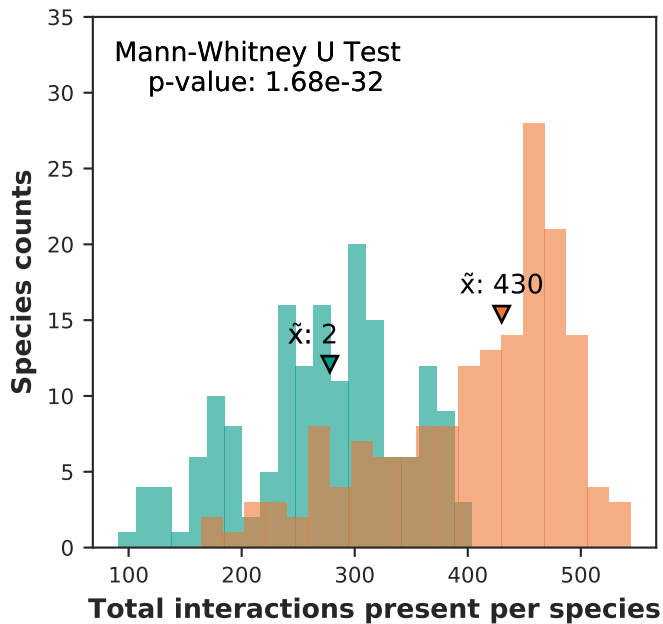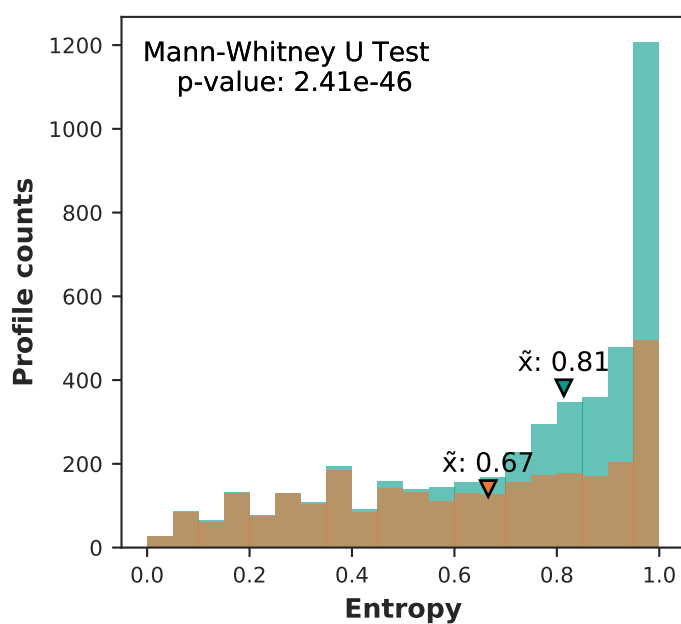**B.**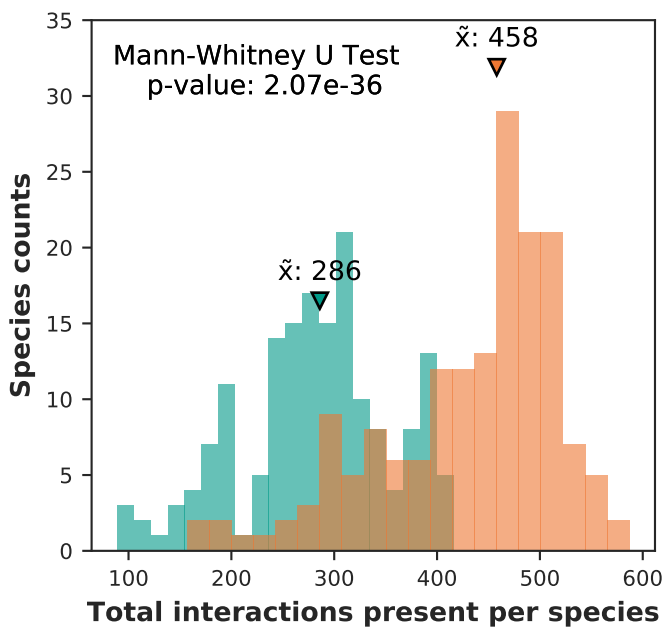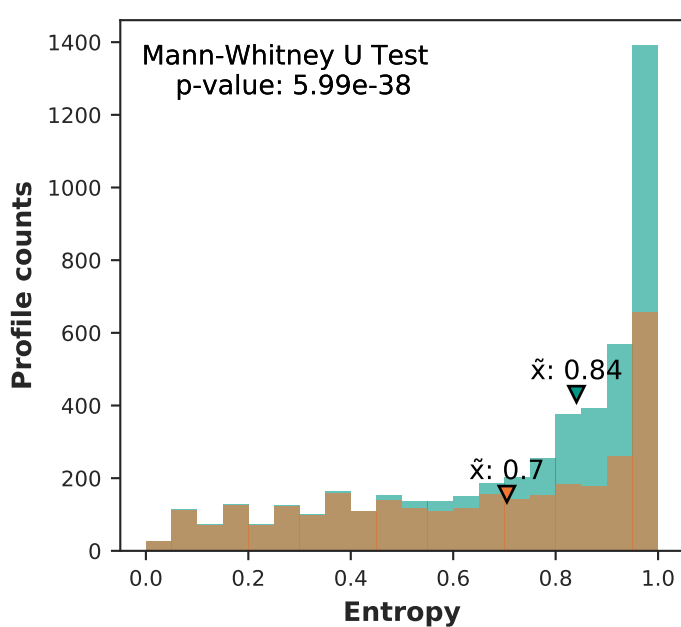

Supplement: S12 Fig — Given for A. Sonicparanoid inferred and B. Broccoli inferred OGs. Median values are presented with the arrows. Mann-Whitney U test shows significant difference between distributions. (PDF) [file pone.0251833.s012.pdf]
