# Supplementary material for: Phylogenetic profiling in eukaryotes: The effect of species, orthologous group, and interactome selection on protein interaction prediction
Source: PLoS One. 2022 Apr 14;17(4):e0251833. doi: 10.1371/journal.pone.0251833 (PMC9009711; doi:10.1371/journal.pone.0251833)
Supplement: S2 Fig — A. BUSCO and Illogical absences as a function of retained LECA OGs in different species. Filled data points are the selected genomes for the prediction accuracy calculations. B. Receiver-operator Curve of two species sets (n = 50) with the most and least BUSCO and illogical absences. The inset gives the Area Under the Curve (AUC) values compared with the random backdrop of 1000 random species sets (violin plot) and the initial species set (teal diamond). (PDF) [file pone.0251833.s002.pdf]

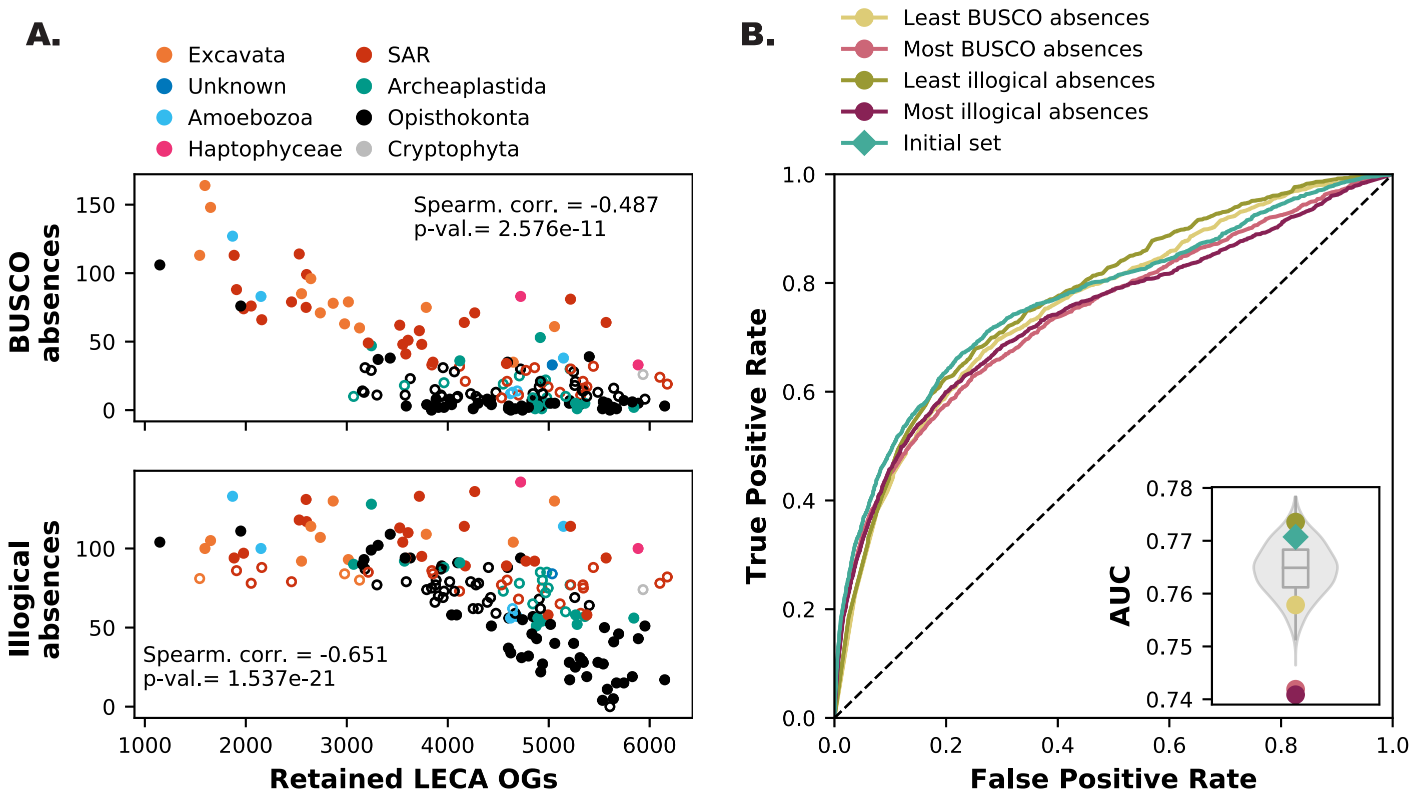
A.
Excavata
SAR
Unknown
Archeaplastida
Amoebozoa
Opisthokonta
Haptophyceae
Cryptophyta
Spearm. corr. = -0.487
p-val.= 2.576e-11
BUSCO absences
Illogical absences
Spearm. corr. = -0.651
p-val.= 1.537e-21
Retained LECA OGs
B.
Least BUSCO absences
Most BUSCO absences
Least illogical absences
Most illogical absences
Initial set
True Positive Rate
False Positive Rate
AUC
