# Supplementary material for: Phylogenetic profiling in eukaryotes: The effect of species, orthologous group, and interactome selection on protein interaction prediction
Source: PLoS One. 2022 Apr 14;17(4):e0251833. doi: 10.1371/journal.pone.0251833 (PMC9009711; doi:10.1371/journal.pone.0251833)
Supplement: S3 Fig — A. The most similar species form more clusters and are overall more similar to each other. B. The most diverse species show no clustering and are overall less similar to each other. C. Receiver-operator Curve of two species sets (n = 50) with the most diverse and most similar species. The inset gives the Area Under the Curve (AUC) values compared with the random backdrop of 1000 random species sets (violin plot) and the initial species set (green diamond). (PDF) [file pone.0251833.s003.pdf]

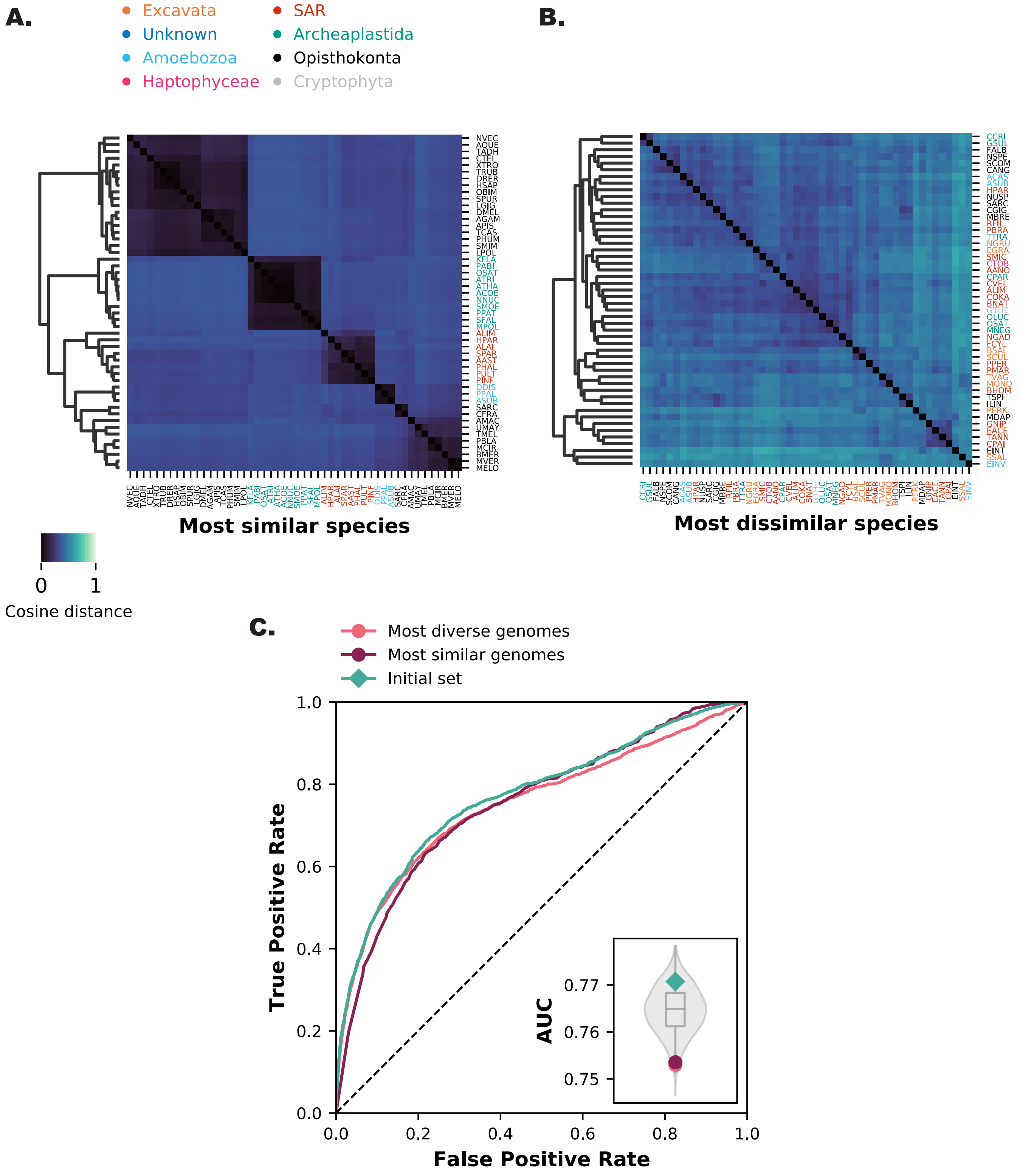
A.
B.
C.
Excavata
Unknown
Amoebozoa
Haptophyceae
SAR
Archeaplastida
Opisthokonta
Cryptophyta
NVEC AQUE TADH CTEL XTRO TRUB DRER HSAP OBIM SPUR LGIG DMEL AGAM APIS TCAS PHUM SMIM LPOL KFLA PABI OSAT ATRI ATHA ACOE NNUC SMOE PPAT SFAL MPOL ALIM HPAR ALAI SPAR AAST PHAL PULT PINF DDIS PPAL ASUB SARC CFRA AMAC UMAY TMEL PBLA MCIR BMER MVER MELO
Most similar species
CCRI GSUL FALB NSPE SCOM CANG ACAS ASUB HPAR NUSP SARC CGIG MBRE RFIL PBRA TTRA NGRU EGRA SMIC CTOB AANO CPAR CVEL ALIM COKA BNAT GTHE OLUC OSAT MNEG NGAD FCYL BSAL SCUL PPER PMAR TVAG MONO BHOM TSPI ILIN PERK MDAP GNIP EACE TANN CPAI EINT SSAL EINV
Most dissimilar species
0
1
Cosine distance
Most diverse genomes
Most similar genomes
Initial set
True Positive Rate
False Positive Rate
0.0
0.2
0.4
0.6
0.8
1.0
AUC
0.75
0.76
0.77
