# Supplementary material for: Phylogenetic profiling in eukaryotes: The effect of species, orthologous group, and interactome selection on protein interaction prediction
Source: PLoS One. 2022 Apr 14;17(4):e0251833. doi: 10.1371/journal.pone.0251833 (PMC9009711; doi:10.1371/journal.pone.0251833)

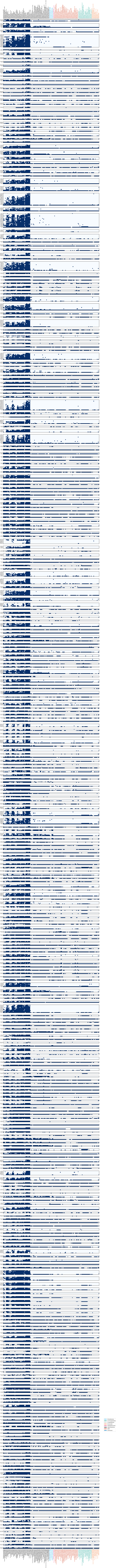

Supplement: S10 Fig — The profiles are sorted according to the species tree. (PDF) [file pone.0251833.s010.pdf]
